# Clinical and Radiographic Evaluation of Procedural Accidents and Errors during Root Canal Therapy

**Published:** 2006-10-01

**Authors:** Mohammad Ali Mozayeni, Mohammad Asnaashari, Seyed Jalil Modaresi

**Affiliations:** 1 *Department of Endodontics, Iranian Center for Endodontic Research, Dental School, Shahid Beheshti University of Medical Sciences, Tehran, Iran*; 2 *Department of Endodontics, Dental School, Shahid Beheshti University of Medical Sciences, Tehran, Iran*; 3 *Department of Endodontics, Dental School, Yazd University of Medical Sciences, Yazd, Iran*

**Keywords:** Endodontic, Procedural Accidents And Errors, RCT

## Abstract

**INTRODUCTION:** Root canal therapy (RCT)_like other dental practices_ can be accompanied with some accidents or unpredictable conditions that are called "procedural accidents".

Having the knowledge about these accidents and their etiology is essential to have RCT completion and to prevent the repeat of these accidents. This study was designed to evaluate accidents occurring during RCT in patients referred to endodontic department of Shaheed Beheshti dental school during 2002.

**MATERIALS AND METHODS:** This study was conducted via descriptive method. Data were collected from observation, clinical examination and oral radiographs, and were recorded in questionnaires, 150 cases from the patients referred to endodontic department were selected randomly and different observed RCT errors were analyzed by SPSS software. The Chi-square and Fisher exact tests were used for analysis.

**RESULTS:** The study showed that 101 patients (67.3%) had one type of RCT errors, and remaining (32.7%) were error free. From the errors studied the most prevalent were "void" which was observed in 41 patients (27.3%), and "ledge" in 39 patients (26%) respectively. The prevalence of other accidents were underfilling in 35 patients (23.3%), poor shaping in 30 patients (20%), overfilling in 23 patients (15.3%), transportation in 13 patients (8.7%), zipping in 3 patients (2%), gouging in 1 patients (0.7%), and strip perforation in 1 patients (0.7%), while no case of broken instruments, vertical fracture, furcation and cervical perforation was observed.

**CONCLUSION:** The most prevalent errors were found in instrumentation and obturation steps, therefore more care and attention must be paid to instructor observation and the education of these stages.

## INTRODUCTION

Endodontics along with other subspecialties in dentistry has enjoyed the same progressive conditions during the recent two decades. This development covers instruments, materials, and theoretical aspects; increasing the long term maintenance chance of root canal treated teeth.

Despite of all these improvements during the time, the concern of inappropriate use is still remained.

RCT procedure, like other dental treatments, may be interrupted by unexpected and unfavorable conditions that are called “Procedural Accidents”. Potential occurrence of procedural accidents in primary steps can necessitate complex treatments and make poor prognosis of RCT (1).

Lots of these problems can be avoided by having acceptable and correct knowledge about the used instruments and suitable treatment plans. Being aware of these accidents and their occurrence leads in to useful treatment and decrease the incidence. One mistake in each step can cause a problem during the following steps of the procedure (1-2). As a result, the study of the prevalence of different procedural accidents, their etiology and other characteristics can help the practitioner to develop improving programs.

The methods for determination of success or failure of treatments are histological and clinical (signs and symptoms) examinations accompany with radiographic observations. It is obvious that histological examination is not as necessary as ethical one. As a result, it can be concluded that clinical findings (signs and symptoms) along with radiographic evaluations are the only possible methods for determination of success or failure in dental procedures (1). After the introduction of X-ray in 1895, dental practitioners did use it as a diagnostic instrument, viewing the result of their treatments (3). In RCT, three radiographs in three steps are necessary; diagnosis, master cone and postobturation (1).

Failure in procedure, despite of exact and overall consideration of related requirements, sometimes occurs. Some factors can affect treatment results which are under and over filling of the root canal, tooth type, the quality and method of obturation, type of medicament, absence or presence of bacteria prior to canal obturation, presence of periapical lesion and etc. Some factors like under and overfilling along with longer treatment period are resulted repeatedly through various studies (1,4).

In order to evaluate the existing situation about the prevalence of the procedural accidents and errors this study was carried out in patients treated in Endodontic Department of Shaheed Beheshti dental school during 2002.

## MATERIALS AND METHODS

The study was descriptive and cross sectional. The technique of observation and clinical and radiographic examinations along with written questionnaire was used for data collection. A total number of 150 patients referred to endodontic department for RCT treatments were selected randomly for the study. The concerned parameters of this study were type and position of the tooth in upper or lower jaws, inclination, calcification, resorption, curve, gouging, furcation perforation, cervical perforation, ledge, transportation, zipping, strip perforation, broken instruments, overfilling, underfilling, void, unsuitable flaring and vertical fracture. The radiographs were examined and the necessary informations regarding the parameters were collected.

The data were analyzed by Chi-square and Fisher exact tests.

## RESULTS

From the cases of this study, 101 patients (67.3%) had one type of errors that were assessed, and 49 patients (32.7%) were without any errors. 74 patients (49.3%) were females and 76 cases (50.7%) were males ranging between 17-47 years old. In 113 cases (75.3%) posterior teeth and in 37 cases (24.7%) anterior teeth were underwent RCT treatment. 81 cases (54%) of the teeth were mandibular and the remaining 69 cases (46%) were maxillary teeth. 22 cases (14.7%) had curved roots, and in 11 subjects (7.3%) tilted tooth was identified. All studied teeth had type I canals.

**Figure 1:**
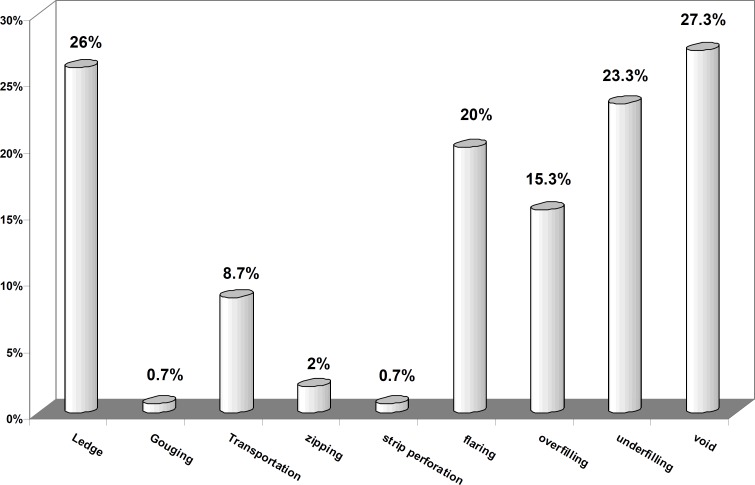
The prevalence of different procedural errors

Among all the errors the most prevalent one was "void" observed in 41 patients (27.3%), and "ledge" in 39 patients (26%). The prevalence of other accidents were: underfilling in 35 patients (23.3%), poor shaping in 30 patients (20%), overfilling in 23 patients (15.3%), transportation in 13 patients (8.7%), zipping in 3 patients (2%), gouging in 1 patients (0.7%), strip perforation in 1 patients (0.7%), and no case of broken instruments, vertical fracture, perforation furcation, cervical perforation was observed (Figure 1).

In 18 (48.6%) cases of anterior teeth, at least one error was observed, while this prevalence was 73.5% (83 cases) in posterior teeth. The observed difference was statistically significant (P<0.005), (Table 1).

64 cases of the accidents were occurred in mandibular teeth while the remaining 37 cases were observed in maxillary teeth. This difference was statistically significant also (P<0.001), (Table 2).

According to root curve the prevalence of errors is classified in Table 3. According to Chi-square test, this difference was statistically significant (P<0.04), (Table 3).

**Table 1:** Prevalence of procedural accidents in anterior and posterior teeth

**Errors**	**with error**	**without error**
**Position**		
Posterior (n=113)	83 (73.5%)	30 (26.5%)
Anterior (n=37)	18 (48.6%)	19 (51.4%)
Total (n=150)	101(67.3%)	49 (32.7%)

Chi-Square test = 7.796,* P* value<0.005

**Table 2:** Prevalence of procedural accidents in maxillary and mandibular teeth

**Errors**	**with error**	**without error**
**Position**		
Maxilla (n=69)	37 (53.6%)	32 (46.4%)
Mandible (n=81)	64 (79%)	17 (21%)
Total (n=150)	101(32.7%)	49 (32.7%)

Chi-Square test = 10.920, *P* value<0.001

**Table 3:** Prevalence of procedural accidents according to root curve

**Errors**	**with error**	**without error**
**Root curvature**		
with curve (n=22)	19 (86.4%)	3 (13.6%)
Without curve (n=128)	82 (64.1%)	46 (35.9%)
Total (n=150)	101(32.7%)	49 (32.7%)

Chi-Square test = 4.245, *P* value<0.04

From the total occurred errors in patients, 7 cases were among teeth with inclination and the remaining 94 cases were observed among teeth without inclination. The difference was not statistically significant (P>0.8). According to apical resorption the existing difference in procedural error occurrence was not considerable and significant (6 cases in teeth with apical resorption and 95 cases in teeth without the problem). Eleven cases of error were also detected in teeth with calcified canals; while 90 cases were occurred in teeth without calcified canals represented no noticeable difference in error prevalence according to canal calcification.

## DISCUSSION

The present study showed that root canal instrumentation and obturation are the most critical steps during which procedural accidents may happen.

The most observed error in this study was related to “void” seen in 41 cases (27.3%). javaheri and Sameri (5) showed that 25% of “underfilling”, 19.14% of “poor shaping”, 21.73% of “void”, 37.5% of “overfilling”, 25% of “apical transportation”, 30% of “ledge formation” and 55.5% of “apical perforation” were subtle for treatment failures. The most prevalent accident was “underfilling” with 25.9%, and “poor shaping” with 25.4%, “void” with 24.8%, “overfilling” with 12.9%, “apical transportation” with 10.8%, “ledge formation” with 5.4%, “apical perforation” with 4.9% and “lateral perforation” with 2.2% had the next orders respectively. The prevalence of poor shaping (20%) and overfilling (15.3%) obtained in the present study is at the range of this study.

A study by Asnaashari (1993) on the errors by dental students showed that “ledge formation” was the most prevalent error while “underfilling” had the next order. “Gouging” was observed with low frequency among the studied errors (6). The prevalence of “ledge formation” found in our study was comparable to the results of this study.

Statistical significant difference was clarified between the errors occurred in teeth with and without curve (P<0.04). Additionally, in 30 cases (23.4%) of straight root canals, and in 9 cases (40.9%) of curved root canals, “ledge formation” was distinguished. Kapalas and Lambriandis have obtained similar finding (25.5% and 56.4% respectively). Their study showed that “ledge formation” was viewed in 51.5% of procedures conducted by dental students while the rate was about 33.2%-40.6% among endodontists. “Ledge formation” in left second molar was repeatedly observed. Their study emphasized on importance of canal curvature in “ledge formation” and showed that 56.4%, 58.2%, and 25.5% prevalence in canals with moderate, severe and straight canals respectively (7). According to the present study, the prevalence of errors in mandibular posterior teeth was more than anterior teeth. The same result was obtained in Kapalas and Lambriandis study, showing the most prevalent error in mandibular second molars (7).

## CONCLUSION

The study revealed less error occurring in access cavity preparation in comparison with instrumentation and obturation stage. This indicates high concern of practitioners' fairly good education level provided in the department for this stage. According to the mentioned studies, “void”, “overfilling and underfilling” as well as “ledge formation” are the errors of more frequency. Most of the errors were observed in instrumentation stage, therefore more care and attention to the education of this stage is very important, and educational observation on students is necessary.
